# Epigenetic Control of Gonadotropin Releasing Hormone Neurons

**DOI:** 10.3389/fendo.2013.00061

**Published:** 2013-05-27

**Authors:** Joseph R. Kurian, Ei Terasawa

**Affiliations:** ^1^Wisconsin National Primate Research Center, University of Wisconsin-Madison, Madison, WI, USA; ^2^Department of Pediatrics, School of Medicine and Public Health, University of Wisconsin, Madison, WI, USA

**Keywords:** gonadotropin releasing hormone, kisspeptin, reproduction, puberty, neuroendocrine, epigenetic, histone, methylation

## Abstract

Epigenetic modifications to the genome, including DNA methylation and histone modifications, occur in response to external stimuli. Reproductive function is highly sensitive to environmental conditions including season, diet, hormonal changes, and exposure to chemical contaminants. GnRH neurons, which play a key role in reproduction, are particularly sensitive to various environmental stimuli. We recently reported that the rhesus monkey GnRH gene exhibits distinct epigenetic changes during embryonic development. More recently, we further found that a similar epigenetic phenomenon occurs across puberty. In this article we highlight recent findings, including those of afferent inputs, to describe the epigenetic control of GnRH circuit development as a link between the environment and reproductive function.

## Introduction

Epigenetic mechanisms are responsible for the tremendous diversity among cell phenotype and function. These mechanisms establish intricate patterns of modifications to DNA and histones, which subsequently control gene expression profiles. Because epigenetic modifications are sensitive to external stimuli, they provide a means for cellular adaptation to environmental pressures. Given the sensitivity of neuroendocrine systems to environmental conditions, significant interest has grown over the epigenetic control of neuroendocrine function.

The past decade has seen an explosion of neuroendocrine epigenetic research, primarily yielding patterns of DNA methylation and histone modifications. To date, how these patterns are established and whether those patterns are critical to neuroendocrine function remains mostly a mystery. However, three reports (Kurian et al., 2010; Iyer et al., 2011; Lomniczi et al., 2013) have recently described epigenetic differences that seem critical to development of the GnRH neuronal circuit, and more importantly appear to pinpoint mechanisms responsible for those epigenetic phenomena. This review synthesizes the findings of those reports to illustrate the promising future of epigenetic research in reproductive neuroendocrine function.

## DNA Methylation in GnRH Neurons

The GnRH neuron is a critical relay in the axis controlling reproductive function. Specifically, its ability to release sufficient, episodic pulses of GnRH peptide into the pituitary portal circulation is essential for gametogenesis in both sexes and ovulation in females. While upstream mechanisms fine-tune GnRH neuronal activity, GnRH neurons also have an intrinsic capacity to release GnRH peptide in a pulsatile manner.

GnRH neurons reside in the preoptic area and base of the hypothalamus, among a complex milieu of other neurons and glia; this presents a challenge for studying the cellular and molecular mechanisms of neuronal differentiation and function. Fortunately, the unique ontogeny of GnRH neurons provides an opportunity to isolate a GnRH neuronal population for *in vitro* studies. In the rhesus monkey, these neurons differentiate from progenitor cells in the nasal placode between embryonic (E) days 32–34 (Ronnekleiv and Resko, 1990; Quanbeck et al., 1997). GnRH neurons subsequently begin migrating into the brain at about E36 and settle down in the hypothalamus by E55 (Terasawa et al., 2001). Isolating the nasal placode after E34 but prior to migration provides a neuronal population that consists of entirely GnRH neurons. We found that placode tissue isolated on E36 developed typical patterns of mature activity (e.g., GnRH peptide release; Terasawa et al., 1993, 1999; Kurian et al., 2010) after about 2 weeks *in vitro*. Wray and colleagues, who developed a similar murine *in vitro* culture model (Fueshko and Wray, 1994), have also reported a period of gradual maturation after isolation from the nasal placode (Constantin et al., 2009). In addition, they report that development of GnRH peptide release patterns is paralleled by increasing GnRH gene expression and peptide biosynthesis (Maurer and Wray, 1997; Moore and Wray, 2000). A question arises. What mechanism triggers increasing gene expression during GnRH neuronal development?

**Figure 1:**
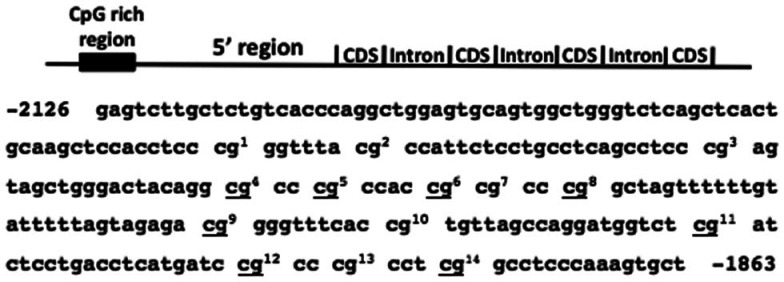
**A Schematic representation of the rhesus monkey *GnRH* gene depicting the location of the 5’ CpG island (CGI) and nucleotide sequence of this region**. This region is the only classifiable CGI within 2500 bases upstream of the *GnRH* gene transcription start site. CpG sites are indicated with numbers corresponding to the CpG sites in Figures 2 and 3.

The genetic control of GnRH gene expression is well characterized and depends on several cis sequences in the 5′ region of the gene. The rat gene contains a neuron specific enhancer region between −1863 and −1571 (Kepa et al., 1992, 1996b; Clark and Mellon, 1995; Whyte et al., 1995). This spans a major region of homology between the rat (−1786 to −1559) and human (−2766 to −2539) genes. Interestingly, this portion of the human gene does not appear to enhance gene expression; in fact, based on serial truncations of the 5′ human GnRH gene in luciferase assay constructs, it appears that this region impedes enhanced gene expression (Kepa et al., 1996a). Importantly, this area has sequence similarity to a 5′ portion of the rhesus monkey GnRH gene. We noticed that this distal 5′ region of the rhesus monkey gene (Figure 1) contains a 243-bp segment (-2126 to -1863) that has 60% GC content and a CpG (cytosine-guanine) dinucleotide observed to expected ratio of 0.65 (14 CpG sites). These characteristics define the region as a CpG island (CGI; Gardiner-Garden and Frommer, 1987). CGIs, when associated with gene promoters, are related to the epigenetic regulation (DNA methylation) of gene expression (Deaton and Bird, 2011).

DNA methylation is the covalent addition of a methyl (-CH_3_) group to nucleotides. Mammalian DNA-methyltransferase (DNMT) enzymes catalyze this reaction, primarily at the 5′ carbon of cytosines in CpG dinucleotides. There are three primary DNMT enzymes (1, 3a, and 3b), each critical to development as demonstrated by embryonic or early postnatal lethality in monogenic null mouse models (Li et al., 1992; Okano et al., 1999). DNMT1 is responsible for faithful maintenance of DNA methylation after replication through cell division (Bestor and Ingram, 1983; Bestor et al., 1988; Hermann et al., 2004). DNMTs 3a and 3b are *de novo* methyltransferases responsible for newly acquired methylation such as during the initial establishment of methylation patterns during early embryonic development (Okano et al., 1998, 1999). Once established, DNA methylation can have several impacts on gene transcription. Methylated DNA can directly alter transcription factor recognition of cis sequences or attract methyl-binding proteins thereby blocking genomic locations from transcription factor assembly. In addition, methyl-binding proteins (e.g., MeCP2, Mbd2, Kaiso) interact with histone modifying factors to alter chromatin structure.

**Figure 2:**
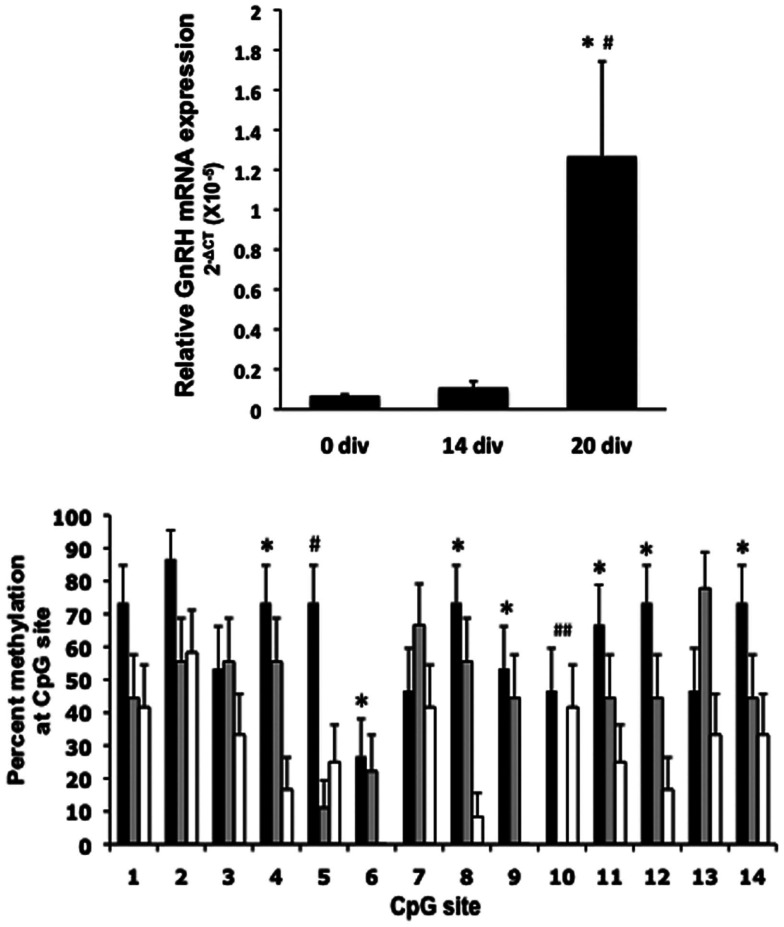
**Changes in GnRH mRNA levels and CGI methylation status during GnRH neuronal development**. Top panel *–* total RNA was extracted from *in vitro* nasal placode cultures at 0, 14, and 20 div (*n* = 4 in all age groups). GnRH mRNA levels, measured by quantitative pcr, started to increase after 14 div, reaching the highest level at 20 div. (*P < 0.05 vs. 0 div; ^#^*P* = 0.05 vs. 14 div). GnRH mRNA levels are relative to 18 s in each sample and analyzed using the ΔCT method. Bottom panel – GnRH neurons dissected from the nasal placode region of two rhesus monkey embryos at E36 and E37 were plated and then harvested on 0, 14, or 20 div. DNA extracted from pooled samples (four cultures) at each time point was bisulfite sequenced. Percent changes in methylation at each CpG site on 0 div (*black bars*), 14 div (*gray bars*), and 20 div (*white bars*) are shown. CpG methylation status was significantly higher at 0 div compared with 20 div at sites 4, 5, 6, 8, 9, 11, 12, and 14 (*P ≤ 0.01). CpG methylation status was significantly higher at 0 div compared with 14 div at site 5 (^#^*P* < 0.05) and at 0 div compared with 14 div but not 20 div at site 10 (^##^*P* < 0.05).

**Figure 3:**
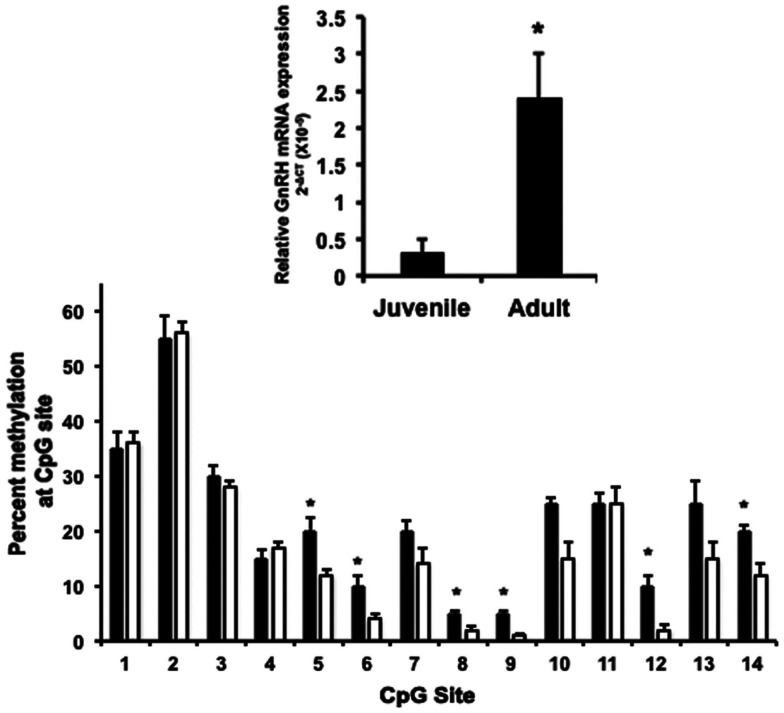
**Changes in GnRH mRNA levels and CGI methylation status across puberty in male rhesus monkey medial basal hypothalamic (MBH) tissue**. Top panel *–* total RNA and DNA was extracted from MBH tissue of juvenile (*n* = 3, mean age 22.7 ± 2.8 months) and adult (*n* = 5, mean age of 110 ± 23.4 months) rhesus monkeys. GnRH mRNA levels, measured by quantitative PCR, were higher in adult compared to juvenile MBH (*P < 0.05). GnRH mRNA levels are relative to 18 s in each sample and analyzed using the ΔCT method. Bottom panel – DNA extracted from the same samples was bisulfite sequenced. Percent changes in methylation at each CpG site for juvenile (*black bars*) and adult (*white bars*) MBH tissue are shown. CpG methylation status was significantly higher in juvenile compared to adult MBH samples sites 5, 6, 8, 9, 12, and 14 (*P ≤ 0.05).

Until our recent studies (Kurian et al., 2010), there were no reports of the epigenetic aspects of GnRH neuron maturation or function. The CGI in the 5′ region of the rhesus monkey *GnRH* gene was suggestive to us that DNA methylation has some role in neuronal function. We hypothesized that increasing peptide release during *in vitro* maturation of GnRH neurons would be related to increased gene expression and changing DNA methylation patterns across the rhesus monkey *GnRH* gene, particularly within the 5′CGI. As suspected, we found that GnRH mRNA levels were low at day 0 but rose dramatically by day 20 of *in vitro* cultures. This increase was paralleled by a dramatic decrease in CpG methylation status in the 5′ CGI (Figure 2). We also found that a comparable phenomenon occurs across puberty. Similar to the previous observation by Plant and colleagues showing that GnRH mRNA levels increase between juvenile and pubertal stages in the medial basal hypothalamus (MBH) of orchidectomized male rhesus monkeys (El Majdoubi et al., 2000), we also observed that GnRH mRNA levels increase in the male MBH across puberty (Figure 3). Furthermore, methylation status of the 5′ CGI of the *GnRH* gene was lower in adult compared to prepubertal male rhesus monkeys (Figure 3; Kurian et al., 2011). This suggests to us that developmental rises in GnRH gene expression are at least partly the result of DNA demethylation across a CGI in the GnRH gene.

When evaluating these findings, it is important to consider the distinct pattern of GnRH neuronal activity across development. In primates, GnRH release is elevated, as indicated by peripheral luteinizing hormone levels, during a brief perinatal period, but then decreases during juvenile development before gradually increasing again through puberty. Our findings suggest that each period of elevated GnRH release is related to demethylation of the *GnRH* gene CGI. Specifically, *in vitro* maturation is representative of embryonic development leading to elevated activity during the perinatal period while measurements in MBH tissue are indicative of developmental changes across pubertal maturation. The decrease in methylation status across puberty is indicative of two potential scenarios. One possibility is that the process initiated in embryonic development might stall during the juvenile period and subsequently resume at puberty onset. Alternatively, the CGI may become re-methylated during juvenile development, and again demethylated at puberty onset. The latter scenario would suggest that lower CpG methylation status must be maintained to enable elevated GnRH gene expression. Consequently, a mechanism responsible for DNA demethylation and perhaps another mechanism responsible for maintaining hypomethylated DNA might both be necessary for the transition to puberty and maintenance of reproductive function. A comparison of CpG methylation status between perinatal and early pubertal MBH tissue will be necessary to differentiate between these two scenarios. Nonetheless, our current findings suggest that DNA demethylation is an important aspect of GnRH neuronal development and function.

## Active DNA Demethylation in GnRH Neurons

Given the postmitotic/non-dividing state of GnRH neurons in our *in vitro* cultures and across the pubertal transition measured in MBH samples, the process of DNA demethylation we discovered must be active. The mechanisms responsible for active DNA demethylation are not well characterized, and until recently skepticism has remained over the existence of this process in mammalian systems.

DNA demethylation is achieved via two general mechanisms, passive or active. Passive demethylation occurs through cell divisions and interruption of maintenance methyltransferase activity. Current pharmaceutical approaches for DNA demethylation target this mechanism. For example, 5-azacytidine (5-aza) is metabolized and subsequently incorporated into DNA, where it then acts as a substrate that covalently traps DNMTs after methyl transfer to the 5-aza nitrogen (Schermelleh et al., 2005; Svedruzic, 2008). Active demethylation is not well characterized. Several mechanisms are proposed, and intense efforts to validate or better characterize these mechanisms continue. For thorough background, we suggest a recent review (Wu and Zhang, 2010) that outlines several promising pathways discovered during the past decade. Presently, a well accepted mechanism is the sequential enzymatic process beginning with oxidation of 5-methylcytosine (5mC) to 5-hydroxymethylcytosine (5hmC), which is carried out by any one of three ten-eleven-translocation enzymes (Tet 1–3). 5hmC is a stable epigenetic modification, though under certain circumstances it is recognized and excised by thymine DNA glycosylases. Base excision repair subsequently completes the transition. Importantly, brain tissue, and particularly the hypothalamus, has the highest reported 5-hydroxymethylcytosine (5hmC) tissue abundance (Branco et al., 2012). In addition, we recently reported that increasing expression of Tet1 and Tet2 across neuronal maturation could influence GnRH gene expression (Kurian and Terasawa, 2012).

Emerging physiological evidence also supports a role for Tet enzymes in neuroendocrine development, and particularly the control of reproductive function. For example, Tet1 knock out mice exhibit deficits in fecundity. This effect is primarily a female specific abnormality, as typical litter sizes result from crossing wild-type females with Tet1 knockout males, whereas wild-type males mated to Tet1 knockout females produces significantly fewer offspring per litter (Dawlaty et al., 2011). A subsequent report suggests this defect in fecundity might be the consequence of abnormal progression of female germ cell development through the second meiotic division prior to ovulation (Yamaguchi et al., 2012). Interestingly, stimulation of the second meiotic division and ovulation requires a GnRH driven LH surge (Mehlmann, 2005). Consequently, given the apparent active DNA demethylation associated with GnRH neuronal development, the effect of Tet depletion on abnormal reproductive function might be the consequence of altered Tet mediated epigenetic differentiation in the neuroendocrine hypothalamus. While hypothalamic expression patterns of Tet enzymes are not yet reported, cortical neuron expression is restricted to Tet2 and Tet3 (Hahn et al., 2013). That this deficit in fecundity was even more pronounced in double knockout (Tet1 and Tet2 knockout) mice (Dawlaty et al., 2013) gives further credence to the suggestion that Tet enzymes are active in the hypothalamus to promote neuronal maturation toward stage specific reproductive function.

As the mechanisms responsible for active demethylation in GnRH neurons are discovered, the ultimate goal will be to characterize how environmental factors influence those epigenetic changes. It should be noted that GnRH neurons experience dramatic shifts in environment during their development and migration. In addition to traveling through or near several tissue types, GnRH neurons are subjected to changing growth factor, chemokine and neurotransmitter levels, some of which form gradients between the nasal region and forebrain (reviewed by Wray, 2010). How these shifts in environment alter the epigenetic landscape in GnRH neurons is yet to be defined, though this background may prove instrumental to the characterization of epigenetic mechanisms pertinent to GnRH neuron maturation.

## Histone Modifications in GnRH Neuronal Function

Histones are an integral component of nucleosomes, the primary units for genome organization or compaction. Consequently, these proteins, through interaction with DNA, have a critical role in determining gene expression patterns. There are four primary classes of histones, 1 through 4. Histones 2 through 4 are components of the core octamer, which DNA circumnavigates in about 146 base pairs to form a single nucleosome. Histone 1 is a scaffold protein, which tightly packages nucleosomes when present. Several variants of this histone are distinguishable by sensitivity to hormones (Banks et al., 2001). Histone 2 also has several variants including A, AX, and B. Histone 2AX is particularly intriguing in the context of neuronal maturation (Lee et al., 2010) and function based on its association with activity dependent DNA double strand breaks in neurons (Suberbielle et al., 2013). Histones 3 and 4 complete the nucleosome octamer with histone 3 the most heavily investigated in the realm of neuroendocrine function. Histone 3 has two variants, H3.3A and H3.3B. These variants are integral to DNA replication independent histone switching, which would be presumed an important mechanism in regulation of post mitotic cell activity. To date, to our knowledge, nothing is reported regarding the relationship between histone switching and hypothalamic neuronal maturation or function.

Histone proteins package DNA largely due to their predominant positive charge attracting negatively charged DNA. Post-translational modifications (PTMs) alter the strength of that attraction, and recruit or repel transcriptional machinery and histone modifying enzymes. Consequently, these PTMs alter gene accessibility and rates of transcription. Several known modifications include acetylation, phosphorylation, methylation, ubiquitination, sumoylation, and glcnacylation. To date, measurements of histone PTMs in neuroendocrine systems have focused on acetylation and methylation. Acetylation leaves a more negative charge on histones and consequently promotes transcription. Methylation is neutral, and depending on the location and degree (mono, di, or tri-methylation), can either promote or inhibit transcription.

Mellon and colleagues were the first to report a pattern of permissive histone modifications enabling elevated or mature GnRH gene transcription (Iyer et al., 2011). Their studies capitalized on the distinct stages of development between two GnRH neuronal cell lines. GN11 cells, originally isolated from a tumor in the mouse nasal placode (Radovick et al., 1991), express GnRH at very low levels, whereas GT1 cells, which were isolated from an analogous tumor in the mouse hypothalamus (Mellon et al., 1990), are characterized by mature activity patterns including elevated GnRH gene expression^1^. In essence, comparisons between these two cell lines are similar to our evaluations of embryonic nasal placode derived neurons from days 0 and 20 *in vitro* described above. They found that the GnRH gene promoter and enhancer regions in immature GN cells were more heavily associated with a repressive histone modification: histone 3 (H3) lysine 9 (K9) di-methylation (me2). On the other hand, the same genomic regions in GT1 cells were associated with the permissive H3K9 acetylation and H3K4me3 PTMs. For comparison, they also evaluated these histone PTM patterns in a non-neuronal (NIH3T3) cell line. The repressive PTMs were high in NIH3T3 cells, intermediate in GN cells and low in GT1 cells. The presence of permissive PTMs was low and similar between NIH3T3 and GN cells but significantly higher in mature GT1 cells.

## Transactivation of Bivalent Domains in Reproductive Neuroendocrine Function

The intermediate repressive chromatin state in GN cells is indicative of a bivalent promoter, where both repressive and permissive histone PTMs maintain genes in a repressed albeit primed position. These chromatin domains were first described in embryonic stem (ES) cells and associated with developmentally regulated transcription factors (Azuara et al., 2006; Bernstein et al., 2006; Pan et al., 2007). Several recent reports, taken together, indicate that establishment of bivalent domains through polycomb repressive complex 2 (PRC2) and mixed lineage leukemia (MLL) is critical for neural lineage differentiation from ES cells (Yu et al., 1995; Yagi et al., 1998; Glaser et al., 2006; Pasini et al., 2007; Shen et al., 2008). The PRC2 component, Ezh2, is responsible for H3K27 methylation, which is subsequently bound by the PRC1 complex to maintain tri-methylation at H3K27. MLL methylates H3K4. Together, these complexes are proposed to establish a bivalent promoter, with the heavily repressive H3K27me3 mark in close proximity to H3K4me. Interestingly, this particular bivalent domain appears related to the juvenile repression and subsequent activation of kisspeptin gene expression during the pubertal transition in female rats (Lomniczi et al., 2013). As a major stimulant of GnRH release, this epigenetic regulation of kisspeptin expression may be instrumental in development of the mature GnRH neuronal circuit.

Similar to the findings of Mellon and colleagues comparing *GnRH* gene promoter structure of immature and mature cell lines, Lomniczi et al. (2013) recently reported that the female rat pubertal transition is accompanied by increased prevalence of activating histone PTMs at the kisspeptin promoter. Specifically, prior to puberty onset, the kisspeptin promoter was associated with a bivalent domain (i.e., H3K27me3 and H3K4me3) and occupied by a component of a polycomb repressive complex, EED. The transition to puberty was accompanied by decreased EED occupancy of the kisspeptin promoter, a gradual loss of the repressive H3K27me3 PTM, and increased levels of the permissive H3K9,14 acetylation and H3K4me3 PTMs. The authors suggest this process is the consequence of increased DNA methylation of the EED promoter, leading to lower EED expression and consequential decreased occupancy of the kisspeptin promoter by the repressive PRC2 (EED, SUZ12, Ezh2) complex. These conclusions were largely based on observations of dramatically delayed puberty in female mice when DNA methylation was inhibited by peripheral administration of the pharmacological DNA-methyltransferase inhibitor 5-azacytidine (5-aza). While the conclusions are consistent with the observations, this 5-aza activity may not be specific to inhibition of post mitotic neuronal DNA methylation. Because the well-characterized mechanism of 5-aza requires nucleoside incorporation into DNA (Schermelleh et al., 2005; Stresemann and Lyko, 2008; Svedruzic, 2008) the mechanism of DNMT inhibition in post mitotic cells by this compound remains unclear. In addition, a significant reduction in growth rate and elevated levels of plasma corticosterone after the initiation of drug treatment (Lomniczi et al., 2013) is indicative of toxicities that likely contribute to delays in maturation. Because of these concerns, more direct approaches (e.g., cell specific genetic or enzyme expression manipulations) will be necessary to clarify the relationship between DNA methylation, chromatin modifications, and puberty onset. Nonetheless, these studies are instrumental to the notion that structural modification of bivalent promoters in the neuroendocrine hypothalamus is an integral step toward puberty onset and reproductive function.

Lomniczi and colleagues’ focused approach toward measuring histone modification status across development at one gene has tremendous value for clarifying the temporal progression of bivalent promoter transactivation. Importantly, they report that permissive histone PTMs (H3K4me3 and H3K9,14 acetylation) accumulate near the kisspeptin promoter during the transition from juvenile to early pubertal stages. This preceded loss of the repressive H3K27me3 PTM, suggesting that recruitment of activating complexes is imperative for transactivation and increased gene expression. Interestingly, a recent discovery points to Tet enzymes as critical mediators of activation at bivalent promoters during neuronal differentiation. Specifically, while Ezh2 (the H3K27 methyltransferase component of PRC2) is critical for progression of neuronal precursor cells toward a neuronal fate, Tet2 or Tet3 appear to complete the process of differentiation. In fact, these studies also found that accumulation of intragenic 5-hydroxymethylcytosine is associated with loss of H3K27me3 near regions with the most significant gene activation during neuronal differentiation. In addition, Tet2 was recently reported to promote H3K4me3 through association with the Set1/COMPASS complex (Deplus et al., 2013). The increasing H3K9,14 acetylation likely indicate RNA Polymerase II associated acetyltransferase (e.g., p300, CBP, PCAF, Gcn5) activity. Though it is possible that this PTM also directs the establishment or maintenance of Tet mediated H3K4 methylation as H3K9,14 acetylation attracts 14-3-3 (Winter et al., 2008), a Tet2 interacting partner (Deplus et al., 2013).

In addition to activation, shifting chromatin structures also enable maintenance or deeper repression at some bivalent promoters across development; for example, neuronal lineage development depends on Jarid1b, which strips H3K4 methylation, consequently repressing bivalent promoters (Schmitz et al., 2011). The histone lysine demethylase, LSD1, has a similar role in chromatin modification, demethylating both di- and mono-methylated H3K4. Importantly, recent preliminary studies (Gill et al., 2012a) found that heterozygous LSD1 knockout female mice exhibit precocious vaginal opening and first ovulation (3–4 days prior to wild-type littermates) with early elevations of plasma gonadotropins and hypothalamic expression of the puberty related gene, tac2 (neurokinin B; Gill et al., 2012b). These findings are analogous to those of Lomniczi et al. (2013), in that lower expression of an epigenetic repressive enzyme is related to elevated hypothalamic expression of a puberty related gene. However, LSD1 expression or activity changes across typical development are currently unknown. In addition, LSD1 also stimulates hormone (ligand associated androgen receptor) mediated gene activation through demethylation of H3K9 (Metzger et al., 2005). Consequently, while preliminary evidence suggests the intriguing possibility that LSD1 directly alters hypothalamic development (including GnRH and kisspeptin neurons) during the pubertal transition, models for cell or region specific genetic manipulations will be necessary to verify this interpretation. These models will be critical for determining the primary activity of LSD1 (i.e., H3K4 or H3K9 demethylation) as it relates to GnRH circuit development and reproductive maturation.

## Conclusion

Our understanding of epigenetic regulation of GnRH neurons and neuroendocrine function in general is in its infancy, though the three reports detailed in this review indicate the importance of a shifting epigenetic structure at genes responsible for the development of reproductive function. Presently, DNA methylation and histone modifications both appear to influence levels of GnRH gene expression through neuronal maturation. On the other hand, kisspeptin gene expression appears most heavily influenced by histone modifications alone in the developing postnatal hypothalamus (Semaan et al., 2012; Tomikawa et al., 2012; Semaan and Kauffman, 2013). Importantly, a similar histone signature (i.e., bivalent domain) seems integral to the development of GnRH and kisspeptin neurons. Specifically, evidence is mounting that activation of a bivalent promoter (Lomniczi et al., 2013) and maintenance of a permissive chromatin state (Iyer et al., 2011) play crucial roles in pubertal maturation and GnRH neuronal function, respectively. Presently, the activating component is not characterized, though evidence points to a Tet enzyme (Kurian and Terasawa, 2012) complex. Decreasing expression or activity of repressive epigenetic enzymes (i.e., PRC2 components or LSD1) may also have a significant role in promoter structure and gene activation in the developing hypothalamus. How these activating and repressive complexes are directed to specific promoters and whether shifts in genomic localization are critical to GnRH circuit development and function remains to be determined. Nonetheless, with these targets to investigate, an understanding of GnRH neuron biology and the means by which an environment modifies neuronal function (i.e., epigenetic modifications), lies at our fingertips.

## Conflict of Interest Statement

The authors declare that the research was conducted in the absence of any commercial or financial relationships that could be construed as a potential conflict of interest.

## Footnotes

^1^The developmental differences in the original GnRH cell lines (GN and GT1) might be the consequence of typical epigenetic differentiation in the 5′ portion of the GnRH gene. Each cell line was derived with a different portion of the GnRH gene promoter driving SV40 expression/tumor initiation. GN cells were generated using a short portion of the human GnRH promoter, which resulted in early tumor production in the placode region, presumably shortly after differentiation from precursor cells. On the other hand, GT1 cells were derived from hypothalamic tumors in animals that expressed SV40 driven by a longer 5′ portion of the rat promoter. This longer promoter contains higher CpG content than the short human promoter used for GN cell generation. Consequently, similar to our proposal that the distal 5′ region of the GnRH gene controls age specific expression of GnRH, developmental epigenetic regulation of the GnRH promoter driven SV40 transgene may underlie the difference in stage of tumor initiation between GN and GT1 cell lines.
